# Long non-coding RNAs in esophageal cancer: molecular mechanisms, functions, and potential applications

**DOI:** 10.1186/s13045-018-0663-8

**Published:** 2018-09-17

**Authors:** Min Su, Yuhang Xiao, Junliang Ma, Deliang Cao, Yong Zhou, Hui Wang, Qianjin Liao, Wenxiang Wang

**Affiliations:** 1 Department of the 2nd Department of Thoracic Surgery, Hunan Cancer Hospital and The Affiliated Cancer Hospital of Xiangya School of Medicine, Central South University, Changsha, 410013 Hunan People’s Republic of China; 2 Department of the Central Laboratory, Hunan Cancer Hospital and The Affiliated Cancer Hospital of Xiangya School of Medicine, Central South University, Changsha, 410013 Hunan People’s Republic of China; 3 Department of Pharmacy, Xiangya Hospital of Xiangya School of Medicine, Central South University, Changsha, 410001 Hunan People’s Republic of China; 4 Department of Thoracic Radiotherapy, Key laboratory of Translational Radiation Oncology, Department of Radiation Oncology, Hunan Cancer Hospital and The Affiliated Cancer Hospital of Xiangya School of Medicine, Central South University, Changsha, 410013 Hunan People’s Republic of China

**Keywords:** Long non-coding RNA, Esophageal cancer, Mechanism, Application, Biomarker

## Abstract

Esophageal cancer (EC) is the sixth leading cause of cancer-related death worldwide. The lack of early diagnostic biomarkers and effective prognostic indicators for metastasis and recurrence has resulted in the poor prognosis of EC. In addition, the underlying molecular mechanisms of EC development have yet to be elucidated. Accumulating evidence has demonstrated that lncRNAs play a vital role in the pathological progression of EC. LncRNAs may regulate gene expression through the recruitment of histone-modifying complexes to the chromatin and through interactions with RNAs or proteins. Recent evidence has demonstrated that the dysregulation of lncRNAs plays important roles in the proliferation, metastasis, invasion, angiogenesis, apoptosis, chemoradiotherapy resistance, and stemness of EC, which suggests potential clinical implications. In this review, we highlight the emerging roles and regulatory mechanisms of lncRNAs in the context of EC and discuss their potential clinical applications as diagnostic and prognostic biomarkers.

## Background

Esophageal carcinoma (EC), a serious malignant cancer, is the sixth leading cause of cancer-related death [1, 2]. Despite advances in multidisciplinary treatment, the 5-year relative survival rate remains less than 20% [3]. EC includes the following two primary pathological types: esophageal adenocarcinoma (EAC) and esophageal squamous cell carcinoma (ESCC) [2]. EAC is the leading histological type observed in patients from western countries, whereas ESCC has become the leading cause of EC in Asian countries and predominates over EAC worldwide [4, 5]. The pathogenesis of EC is complex and differs between EAC and ESCC. For EAC, the primary predisposing cause is metaplasia that is likely caused by chronic exposure to acid and bile reflux, such as in the case of Barrett’s esophagus and chronic gastroesophageal reflux disease [6]. However, the origin of ESCC carcinogenesis is not fully understood. Early-stage EC can be effectively treated with curative surgery, but for advanced cases, the therapeutic strategies are limited [7]. Unfortunately, EC patients are usually diagnosed at an advanced stage accompanied with lymphatic metastasis, and therefore they are not eligible for surgical resection [8]. The current standard treatment for these patients is concurrent definitive chemo- or radiotherapy, or a combination of both [9]. However, therapy resistance and tumor recurrence are major obstacles for EC therapy and are critical issues leading to poor prognoses [10]. Within the EC, a small number of cells termed cancer stem-like cells (CSCs) are considered to account for the initiation, recurrence, and therapeutic resistance of EC [10]. In recent years, compelling evidence has demonstrated the crucial roles of long non-coding RNAs (lncRNAs) in the pathogenesis and progression of EC.

LncRNAs, which are an emerging focus of current cancer research, are defined as endogenous cellular RNAs that are more than 200 nucleotides in length and are incapable of encoding protein [11, 12]. Initially, lncRNAs were considered as transcriptional “noise,” given their relatively low expression levels compared with mRNAs and their lack of protein-coding capacity [13]. However, in-depth studies in recent years revealed that lncRNAs possess certain characteristics of mRNAs; for instance, lncRNAs are transcribed by RNA polymerase II, equipped with a 3′ polyA tail and a 5′ cap, and contain a promoter and structure consisting of multiple exons [14, 15]. Accumulating evidence suggests that the aberrant lncRNA expression is associated with oncogenesis and the development of various cancers [16, 17]. LncRNAs have been shown to interact directly with DNA, RNA, and proteins to regulate several mechanisms, including the following: chromatin modification, RNA transcription, pre-mRNA splicing, mRNA translation, and other mechanisms that influence gene expression [18, 19]. Moreover, several lncRNAs have been functionally well-characterized in cancer pathogenesis and development and may be potential novel biomarkers for cancer diagnosis and prognosis, as well as therapeutic targets.

In this review, we focused our efforts on the recent findings regarding the molecular mechanisms and functional roles of lncRNA in EC oncogenesis and development. In addition, we discussed the potential implications of lncRNAs as biomarkers for the diagnosis and prognosis of EC.

## Mechanisms of lncRNAs in EC

**Fig. 1:**
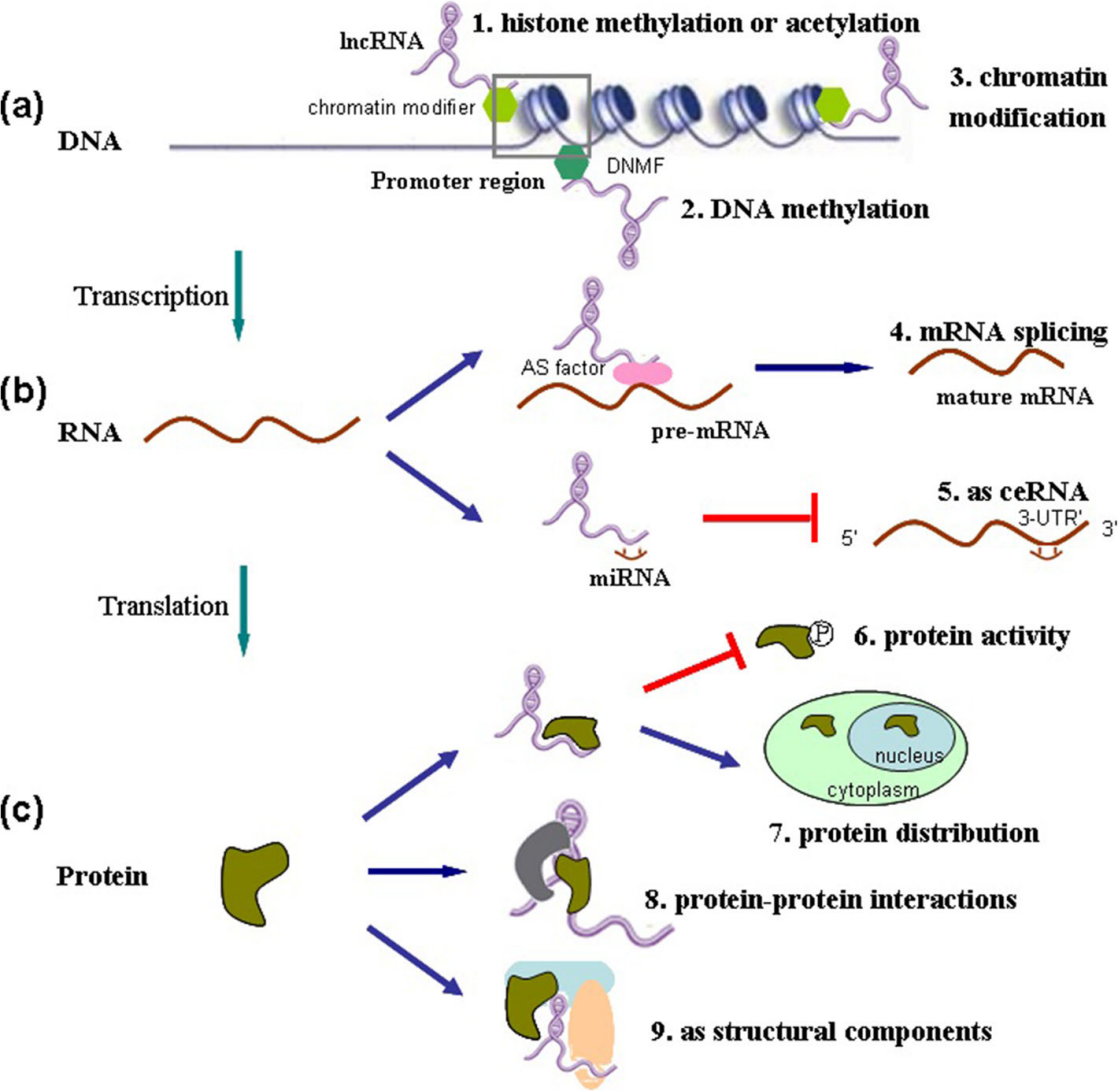
The molecular mechanisms underlying esophageal cancer-related lncRNAs rely on interactions with cellular macromolecules. (a) LncRNAs localize to the chromatin. LncRNAs recruit chromatin modification complexes to the promoter region of chromatin and the results in (1) histone methylation or acetylation, (2) DNA methylation; lncRNAs recruit chromatin modification complexes to specific loci of chromatin and modulate gene expression through (3) chromatin modification. (b) LncRNAs interacts with RNA. (4) LncRNAs interacts with pre-mRNA, affect alternative splicing and help to produce mature mRNAs; (5) lncRNAs act as miRNA sponges or compete for endogenous RNAs (ceRNAs) and compete for miRNAs to inactivate these small regulatory RNAs, followed by relief of the repression of the target gene. (c) LncRNAs interact with proteins.( 6) LncRNAs regulates protein dephosphorylation and activity; (7) lncRNAs regulate protein localization; (8) lncRNAs modulate protein–protein interactions; (9) lncRNAs directly localize within cellular compartments to serve as structural components

LncRNAs may act as signals or guides for the recruitment of chromatin-modifying complexes to induce transcription, and they may even act as decoys that bind to transcription factors (TFs) to prevent the transcription factors from binding to target gene promoter regions, thereby suppressing transcription [20, 21]. In addition, lncRNAs can hybridize to pre-mRNAs, block the recognition of splice sites by spliceosomes, and regulate the alternative splicing of pre-mRNAs to produce alternate transcripts [17, 22]. An additional biological function of lncRNAs may include serving as “miRNA sponges” through interactions with miRNAs to inactivate these small regulatory RNAs and hence increase the expression of the miRNA target genes [23–25]. Finally, lncRNAs may be involved in the modulation of protein localization, activity, and function [26]. In this section, we highlight the molecular mechanisms of lncRNAs in EC via their interactions with chromatin, DNA, RNA, and regulatory proteins (Fig. 1 and Table 1).

**Table 1:** Molecular mechanisms of deregulated lncRNAs in EC

Mode of mechanism	LncRNA	Expression	Molecular mechanisms	Ref.
Localizes to chromatin	CASC9	Up	Negatively regulates PDCD4 expression through recruiting EZH2 and altering H3K27me3 level	[32]
	SBF2-AS1	Up	Binds with PRC2 and guided PRC2 to the promoter of CDKN1A and decreased CDKN1A expression	[33]
	CASC9	Up	Activates the FAK-PI3K/Akt signaling pathways through upregulating LAMC2 expression by interacting with the CREB-binding protein	[35]
	LUCAT1		Binds to DNMT1 and regulates its stability, inhibits the expression of tumor suppressors through DNA methylation	[41]
	NMR,		Interacts with BPTF and recruits it to chromatin, upregulates expression of MMP3 and MMP10 via ERK1/2 activation	[42]
Interacts with RNA (mRNA splicing)	LincRNA-uc002yug.2	Up	Associates with alternative splicing of RUNX1	[48]
Interacts with RNA (ceRNA)	ATB	Up	Regulates miR-200b/Kindlin-2 axis	[51]
	SNHG16	Up	Regulates miR-140-5p/ZEB1 axis	[54]
	HOTAIR	Up	Regulates miR-125/HK2 and miR-143/HK2 axis, miR-148a/Snail2 axis, miR-1/CCND1 axis	[61] [64] [65]
	CCAT1	Up	Regulates miR-7/HOXB13 axis	[69]
	NEAT1	Up	Regulates miR-129/CTBP2 axis	[70]
	PVT1	Up	Regulates miR-203/LASP1 axis	[71]
	SNHG1	Up	Regulates miR-338/CST3 axis	[72]
	UCA1	Up	Regulates miR-204/ Sox4 axis	[73]
	XIST	Up	Regulates miR-101/EZH2 axis	[74]
	TUSC7	Down	Regulates miR-224/DESC1 axis	[75]
Interacts with protein	LINC01503	Up	Activates ERK signaling via MAPK and increases AKT signaling	[78]
	EZR-AS1	Up	Upregulates EZR expression by causing SMYD3 redistribution	[85]

## LncRNAs localized to the chromatin

LncRNA-dependent chromatin regulation involves the recruitment and modulation of histone-modifying enzymes that induce chromatin modification at promoters and enhancers [27, 28]. In this manner, lncRNAs can regulate gene expression through histone modification, DNA methylation, and chromatin structure alteration [29, 30].

It has been demonstrated that many lncRNAs are associated with polycomb repressive complex 2 (PRC2), which is responsible for the trimethylation of lysine 27 on histone 3 (H3K27me3) and mediates the silencing of the target gene through local chromatin reorganization [31]. Enhancer of zeste homolog 2 (EZH2) and SUZ12 are subunits of the PRC2 complex. Wu et al. [32] demonstrated that cancer susceptibility candidate 9 (CASC9) downregulates the expression of PDCD4 via the recruitment of EZH2 to alter H3K27me3 levels at the promoter region of PDCD4. In addition, SET-binding factor 2 antisense RNA1 (SBF2-AS1) was demonstrated to bind to SUZ12 and guide PRC2 to the promoter of CDKN1A to decrease CDKN1A expression in ESCC [33].

The acetylation of histone H3 and H4 is another core mechanism through which chromatin structure and gene expression are altered [34]. In addition to recruiting EZH2, CASC9 also associates with the transcriptional coactivator CBP in the nucleus to increase the enrichment of CBP and H3K27 acetylation in the promoter region of LAMC2, thereby increasing LAMC2 expression [35].

DNA methylation is one of the most common and stable chromatin modification that is associated with gene inactivation [36, 37]. Lung cancer associated transcript 1 (LUCAT1) was originally identified in smoking-related lung cancer [38] and is also associated with colorectal cancer [39], clear cell renal cell carcinoma, and osteosarcoma [40]. A recent study demonstrated that LUCAT1 binds to DNMT1, the most abundant DNA methyltransferase in mammalian cells, and regulates its stability by inducing the ubiquitination of DNMT1 in ESCC [41]. The high levels of LUCAT1 in ESCC inhibit the expression of certain tumor suppressors through DNA methylation.

In addition, NMR, a novel lncRNA identified through microarray assays, was found to be upregulated in ESCC tissues and primarily located in the cell nucleus [42]. NMR interacts with the chromatin regulator BPTF [42], which was demonstrated to be involved in ATP-dependent chromatin remodeling and transcriptional regulation [43]. Hence, by recruiting BPTF to specific loci of chromatin, NMR upregulates the expression of MMP3 and MMP10 via ERK1/2 activation to promote ESCC tumorigenesis.

## LncRNAs target RNA

Following the transcription of RNA in the nucleus, a series of conserved processes are essential for the production of mature mRNAs that can be translated into proteins. LncRNAs modulate gene expression at the RNA level through the regulation of alternative splicing and the stability of mRNAs; additionally, lncRNAs act as miRNA sponges or competing endogenous RNAs (ceRNAs) [17, 26].

### mRNA splicing

Alternative splicing is a regulated process that produces different mRNA splice isoforms from a single mRNA precursor [44]. Alternative splicing produces different proteins that are translated from alternatively spliced mRNAs. This process results in proteins that have different biological functions and phenotypes [45, 46]. LincRNA-uc002yug.2, a lncRNA principally localized to the nucleus, is increased significantly in ESCC tissues [47]. LincRNA-uc002yug.2 was shown to promote the recruitment of alternative splicing factors and RUNX1 to the nucleus to produce more RUNX1a (an inhibitor of RUNX1) relative to the other two isoforms (RUNX1b and RUNX1c) [48]. Moreover, decreased RUNX1 expression was shown to reduce the mRNA levels of CEBPα, which promotes cell proliferation [49]. Thus, LincRNA-uc002yug.2 may modulate cell proliferation and the tumor growth of ESCC through the alternative splicing of RUNX1.

### CeRNA

The ceRNA hypothesis is a novel theory regarding the regulation of gene expression through post-transcriptional processes [50]. According to this hypothesis, ceRNA acts as a molecular sponge for common miRNAs through the miRNA response elements (MREs) to regulate the expression of the target genes of the miRNAs. Several lncRNAs have recently been found to act as ceRNAs by sponging miRNAs to reduce their inhibitory effect on their target protein-coding mRNAs.

There are numerous examples of lncRNAs functioning as sponges and therefore oncogenes in EC. Transforming growth factor β (ATB) may act as a ceRNA of miR-200b and thereby promote the expression of kindlin-2 in ESCC, as miR-200b potentially targets the 3′-untranslated region (3′-UTR) of kindlin-2 [51]. Kindlin-2 was reported to act as an oncogene by participating in cytoskeleton shaping via RhoA/FAK signaling to modulate cell migration [52, 53]. Moreover, ATB is overexpressed in ESCC. The knockdown of ATB resulted in the suppression of activated RhoA and phosphorylated FAK and the inhibition of ESCC cell proliferation, migration, and lung metastasis. Hence, the dysregulation of the lnc-ATB/miR-200b/kindlin-2 axis is involved in the development of ESCC. Small nucleolar RNA host gene 16 (SNHG16) is significantly upregulated in ESCC, and this lncRNA is primarily distributed within the cytoplasm [54]. SNHG16 promotes the progression of ESCC cells by binding with miR-140-5p to positively regulate the miR-140-5p target gene ZEB1. The transcription factor ZEB1 has been reported to promote the epithelial-to-mesenchymal transition (EMT) in multiple tumors, including ESCC [55, 56]. Thus, SNHG16 functions as an oncogene by promoting tumor progression by competing with miR-140-5p to regulate ZEB1.

HOX transcript antisense RNA (HOTAIR) is a well-studied lncRNA that was shown to have multiple ceRNA regulatory roles in EC. HOTAIR is transcribed from the antisense strand of the HOXC gene cluster [57] and has been shown to be involved in reprogramming chromatin organization and promoting cancer cell proliferation and metastasis [58–60]. Ma et al. [61] reported that HOTAIR upregulates the expression of HK2 by functioning as a molecular sponge for miR-125 and miR-143, both of which modulate HK2 expression by targeting the 3′UTR of HK2. HK2 is overexpressed in a variety of cancers and is well-known to play a key role in tumor growth and metastasis [62, 63]. Hence, HOTAIR plays an oncogenic role in ESCC. Another study by Xu et al. [64] showed that HOTAIR promoted EC cell invasion and metastasis by promoting the EMT through the upregulation of Snail2, a transcription factor associated with the EMT. Mechanistically, HOTAIR positively regulates Snail2 by sponging miR-148a. HOTAIR has also been reported to bind directly to miR-1 and act as an endogenous sponge to inhibit miR-1 expression [65], thereby positively regulating CCND1 expression. CCND1 functions as an oncogene in various human cancers by promoting G1-S progression to regulate the cell cycle [66–68]. Thus, the HOTAIR/miR-1/CCND1 axis may promote ESCC tumorigenesis.

Several other lncRNAs have also been shown to function as oncogenes through sponging miRNAs and positively regulating their target tumor-promoting genes, including CCAT1 [69], NEAT1 [70], plasmacytoma variant translocation 1 (PVT1) [71], small nucleolar RNA host gene 1 (SNHG1) [72], UCA1 [73], and XIST [74].

In addition to these oncogenic lncRNAs sponges, there are also lncRNA sponges involved in tumor suppression. Tumor suppressor candidate 7 (TUSC7) is downregulated in ESCC tissues and is associated with shorter OS time in ESCC patients [75]. TUSC7 was shown to bind to and negatively regulate the expression of miR-224, which specifically binds to the 3′UTR region of DESC1 to negatively regulate DESC1 expression. DESC1 is an epithelial-specific enzyme and exerts tumor suppressive roles by promoting cell apoptosis via the downregulation of the EGFR/AKT pathway in ESCC [76, 77]. Thus, TUSC7 promotes cell apoptosis and suppresses the proliferation and chemotherapy resistance of ESCC cells by regulating the DESC1/EGFR/AKT pathway through miR-224. These findings indicate that TUSC7 may act as a tumor suppressor in ESCC.

## LncRNAs interact with proteins

Several lncRNAs have been reported to interact with specific proteins to participate in global cellular processes in EC by regulating protein activity and function, modulating protein–protein interactions or directing the localization of proteins within cellular compartments to serve as structural components [26].

Through RNA pull-down assays and chromatin isolation by RNA purification (ChIRP), Xie and colleagues [78] demonstrated that LINC01503 could bind with both EBP-1 and ERK2 in the cytoplasm. Further analysis revealed that both basal and EGF- and IGF-induced phosphorylation of ERK1/2, Akt, p70S6K, and mTOR were significantly decreased following the knockdown of LINC01503. In addition, silencing LINC01503 expression increased the binding of EBP-1 to the PI3K subunit p85, suggesting that LINC01503 inhibits PI3K deubiquitination to activate the PI3K/Akt signaling pathway. Taken together, these findings suggest that LINC01503 contributes to ESCC cell proliferation, migration, and invasion through the activation of the ERK/MAPK and PI3K/Akt signaling pathways.

Snail, an important transcription factor influencing the EMT, binds to the E-box site in the promoter region of E-cad to suppress E-cad expression [79, 80]. This suppression triggers the EMT in a variety of cancer types, including ESCC [81, 82]. Thus, the nuclear localization of Snail is crucial for its role in the EMT progression [83]. A study by Zhang et al. [84] showed that Sprouty4-Intron 1 (SPRY4-IT1) directly increased the transcription and expression of Snail, as well as its nuclear localization, by directly binding with Snail in ESCC cells. SPRY4-IT1 is highly expressed in ESCC tissues, and overexpression of SPRY4-IT1 promotes the EMT in ESCC cells. This finding demonstrates that SPRY4-IT1 may act as an oncogene in ESCC progression via the regulation of Snail.

EZR-antisense 1 (EZR-AS1) interacts with and is part of the RNA polymerase II complex [85]. RIP assays have revealed that EZR-AS1 directly binds with SMYD3, a histone H3-lysine 4 (H3K4)-specific methyltransferase, causing SMYD3 redistribution and recruiting SMYD3 to the binding site in GC-rich regions downstream of the EZR promoter in ESCC cells. This recruitment results in the localized enrichment of SMYD3 and H3K4me3 in the EZR promoter. Lastly, EZR-AS1 was shown to promote ESCC cell migration via enhancing EZR transcription and expression.

## Functions of lncRNAs in EC

Increasing evidence in the last decade indicates that lncRNAs function in a plethora of biological processes, including cell survival and apoptosis, cell cycle progression and proliferation, migration and invasion, stemness, and chemoradiotherapy (CRT) resistance (Table 2).

**Table 2:** Functions of deregulated lncRNAs in EC

Function	LncRNA	Expression	Targets	Ref.
Sustaining growth signaling	LINC00152	Up	EGFR	[89]
Evading growth inhibitors	AK001796	Up	MDM2/p53 signaling	[93]
	CASC2	Down	miR-18a-5p/PTEN axis	[99]
Uncontrolled replicative immortality	CDKN2B-AS1	Up	hTERT	[104]
	BC032469	Up	hTERT	[105]
Activating invasion and metastasis	PVT1	Up	–	[106]
	SNHG16	Up	miR-140-5p/ZEB1 axis	[54]
	HOTAIR	Up	miR-148a/Snail2 axis	[64]
	SNHG1	Up	Notch pathway	[112]
	MALAT1	Up	Ezh2-Notch1 signaling, miR-200a/ZEB1, and miR-200a/ZEB2 axis	[113] [114]
	CASC9	Up	–	[115]
	GHET1	Up	–	[116]
	TTN-AS1	Up	miR-133b/Snail1 axis, miR-133b/FSCN1 axis HuR	[117]
	HOTTIP	Up	miR-30b/HOXA13 axis	[118, 119]
Promoting angiogenesis	HNF1A-AS1	Up	VEGF	[123]
Resisting apoptosis	TP73-AS1	Up	BDH2	[126]
	POU6F2-AS2	Up	Ybx1	[127]
	AFAP1-AS1	Up	–	[128]
	LET	Down	–	[129]
Chemoradiotherapy resistance	AFAP1-AS1	Up	–	[132]
	LOC285194	Up	–	[136]
	BOKAS	Up	WISP1	[47]
	TUSC7	down	miR-224/DESC1	[75]
Regulation of EC stem cells	MALAT1	Up	OCT4 and Nanog	[147]

## Involvement of lncRNAs in the hallmarks of cancer

Although cancer is a complex and heterogeneous disease, one of the common features of cancer is that abnormal cells grow beyond control. In 2000, Hanahan and Weinberg proposed six properties that are the hallmarks of cancer [86]. These basic hallmarks include sustaining growth signaling, evading growth inhibitors, uncontrolled replicative immortality, tissue invasion and metastasis, promoting angiogenesis, and resisting cell death.

### Sustaining growth signaling

Tumor cells acquire the capability to sustain growth signaling through autocrine and paracrine growth factor pathways [87]. LncRNAs mediate tumor growth signals primarily by acting on the regulation of growth factors or receptors. Epidermal growth factor receptor (EGFR) is a crucial regulator in tumor growth [67]. LINC00152 has been reported to directly bind to EGFR and activate the downstream PI3K/AKT signaling pathway in gastric cancer [88]. Recently, Yang and colleagues [89] demonstrated that both LINC00152 and EGFR were highly expressed in the subtype 1 of ESCC. By performing differential coexpression analysis (DCEA) and traditional differential expression analysis (DEA), the authors detected the “gain” of miRNA-mediated crosstalk between EGFR and LINC00152 in ESCC. However, the exact regulatory relationship between LINC00152 and EGFR needs further clarification.

### Evading growth inhibitors

Several tumor suppressors that regulate the cell cycle and inhibit cellular growth have been discovered, such as p53 and PTEN [87]. Certain lncRNAs regulate EC cell growth through altering the expression of these tumor suppressors. P53 is a master “gatekeeper” of the cell and functions as a tumor suppressor gene [90]. P53 regulates the expression of numerous target genes, which leads to the suppression of tumor growth through the induction of cell cycle arrest and apoptosis. Mouse double minute 2 (MDM2), which acts as a primary regulator of p53, inhibits the transcription of p53 via promoting its ubiquitination and degradation [91, 92]. Thus, the MDM2/p53 axis is an important signaling pathway that regulates cell growth and the cell cycle. The expression of lncRNA AK001796 was shown to be positively associated with MDM2 levels in ESCC tissues [93]. Knockdown of AK001796 downregulated the expression of MDM2 and upregulated the expression of p53 along with its target gene, p21. Taken together, these findings indicate that AK001796 mediates the cell cycle and cell proliferation by activating p53 signaling.

Another important tumor suppressor is PTEN, which is a crucial inhibitor of the PI3K/AKT/Mtor pathway [94, 95]. This signaling pathway is a well-known regulator of the cell cycle, proliferation, migration, and apoptosis [96–98]. Zhang et al. [99] reported that miR-18a-5p directly binds to the 3′UTR regions of PTEN, thereby inhibiting the expression of PTEN in EC cells. In addition, lncRNA cancer susceptibility candidate 2 (CASC2) was demonstrated to directly interact with miR-18a-5p and modulate the expression of PTEN by targeting miR-18a-5p. These data revealed that CASC2 may inhibit the proliferation of EC cells.

### Uncontrolled replicative immortality

The telomeres, located at the chromosome ends, are important for limiting cell division cycles and replication. Telomerase was shown to regulate the expression of a variety of growth-controlling genes and promote cell proliferation [100, 101]. As a catalytic subunit of telomerase, human telomerase reverse transcriptase (hTERT) maintains the telomere length and plays crucial roles in cell proliferation [102, 103]. Hu et al. [104] reported that hTERT expression is mediated by lncRNA cyclin-dependent kinase inhibitor 2B-antisense 1 (CDKN2B-AS1). Thus, the knockdown of CDKN2B-AS1 rescued the slow proliferation of EC109 cells induced by β-elemene, an anticancer drug. BC032469, another lncRNA that is overexpressed in ESCC tissues, was positively associated with a larger tumor size and shorter OS [105]. Silencing BC032469 expression in ESCC cells resulted in the inhibition of cell proliferation. Mechanical assays revealed that BC032469 induced cell cycle arrest in the G0/G1 phase by regulating the expression of hTERT.

### Activating invasion and metastasis

The process of EMT has been confirmed to play a critical role in cell invasion in various types of cancer. This process transforms adherent and polarized epithelial cells into invasive and motile mesenchymal cells, accompanied with the loss of epithelial markers E-cadherin and the acquisition of mesenchymal markers N-cadherin and vimentin. Multiple lncRNAs have been demonstrated to be involved in EC development through the regulation of the EMT and metastasis. PVT1 has been identified as an oncogene, and high PVT1 expression was shown to be associated with the development of EC. Upregulation of PVT1 in EC cells resulted in increased N-cadherin and vimentin expression and decreased E-cadherin expression [106]. Thus, PVT1 induced the EMT and promoted the invasion of EC cells.

The EMT is also induced by several signaling pathways, such as the TGF-β and Notch signaling pathways [107–109]. The Notch signaling pathway is important for the development and progression of some tumors [110, 111]. The lncRNA SNHG1 was shown to be overexpressed in ESCC tissues and correlated with lymph node metastasis, depth of invasion, and shorter OS time in ESCC patients [112]. Silencing the expression of SNHG1 in ESCC cells was demonstrated to inhibit cell proliferation and cell invasion capacity, as well as the EMT phenomenon, through suppressing the Notch signaling pathway.

Additional lncRNAs involved in the EMT and invasion of EC include SNHG16 [54], HOTAIR [64], SNHG1 [112], metastasis associated in lung adenocarcinoma transcript 1 (MALAT1) [113, 114], CASC9 [115], gastric carcinoma highly expressed transcript 1 (GHET1) [116], TTN-antisense 1 (TTN-AS1) [117], and HOXA transcript at the distal tip (HOTTIP) [118, 119].

### Promoting angiogenesis

Angiogenesis is a universal characteristic of EC progression, as it supplies the tumor with nutrients and oxygen and facilitates proliferation and migration [120, 121]. Vascular endothelial growth factor (VEGF) is the most potent activator of angiogenesis [122]. LncRNAs may regulate angiogenesis primarily by regulating VEGF. HNF1A-antisense 1 (HNF1A-AS1) is the sole lncRNA reported to modulate VEGF thus far. Recently, Wang reported that the knockdown of HAS1 suppressed the expression of VEGF in ESCC cells [123]. However, direct supporting evidence that HAS1 inhibits angiogenesis requires further studies.

### Resisting cell death

The following three major pathways lead to cell death: apoptosis, autophagy, and necrosis [124]. Currently, few lncRNAs are known to be associated with the latter two pathways of cell death in EC, but several lncRNAs are involved in apoptosis via regulating the transcription of key apoptotic factors. For instance, BDH2, which functions as an anti-apoptotic factor, is regulated by survivin via the caspase-3-independent pathway [125]. P73 antisense RNA 1T (TP73-AS1), a lncRNA mapped to chromosome 1p36.32, was shown to mediate apoptosis via BDH2 [126]. The knockdown of TP73-AS1 suppressed BDH2 expression and induced the expression of pro-apoptotic proteins, which subsequently induced apoptosis in EC cells. POU6F2-antisense 2 (POU6F2-AS2) is a lncRNA that is especially overexpressed in ESCC tissues and cells other than EAC [127]. POU6F2-AS2 knockdown induced prolonged DNA tails in ESCC cells following ionizing radiation (IR) and caused sensitivity to IR, indicating that POU6F2-AS2 is involved in the DNA damage response. Mechanical assays revealed that POU6F2-AS2 interacts with DNA repair-related protein Ybx1 and mediates the recruitment of Ybx1 to the promoter region of target genes, such as p53 and CCNB1. Finally, the dysregulation of POU6F2-AS2 expression in ESCC cell lines regulates cell survival after IR. However, the exact underlying mechanism of several other lncRNAs involved in apoptosis of EC cells, such as AFAP1-AS1 [128] and Low Expression in Tumor (LET) [129], warrants further investigation.

## LncRNAs related to chemoradiotherapy resistance

Acquired CRT resistance is one of the major obstacles in the treatment of EC [130]. Studies have shown that less than 50% of patients benefit from CRT treatment, and the remaining half patients present resistance to CRT [131]. Recently, several lines of evidence have suggested that lncRNAs are likely to play vital roles in CRT resistance in EC. Zhou et al. [132] examined 18 lncRNAs that were previously reported to be dysregulated in EC or involved in CRT resistance in cisplatin-resistant ESCC cell lines and samples from patients treated with dCRT. The authors detected that three lncRNAs (AFAP1-AS1, UCA1, and HOTAIR) were dysregulated in cisplatin-resistant cells compared with the parent cell line. Moreover, AFAP1-AS1 was significantly overexpressed in tumor tissues compared to the adjacent paired tissues. Furthermore, the overexpression of AFAP1-AS1 was strongly related to the response to dCRT and to the shorter progression-free survival (PFS) and OS of ESCC patients. High AFAP1-AS1 expression could predict resistance to CRT in patients with ESCC. Another lncRNA, LOC285194, also known as LSAMP antisense RNA 3, has been reported to be downregulated in several cancers, including EC and was found to be closely associated with a poor patient prognosis [133–135]. Additionally, the low expression of LOC285194 could predict resistance to CRT [136]. As mentioned above, TUSC7 promotes cell apoptosis and inhibits chemotherapy resistance through the miR-224-dependent regulation of DESC1 [75].

Tumor radioresistance is very complex and heterogeneous. Although the mechanism underlying radioresistance is not well-understood, several signaling pathways have been demonstrated to be involved in radioresistance. The Wnt/β-catenin pathway is well-known to promote cell growth and survival and has been proven to modulate radioresistance in various cancers [137, 138]. WISP1, a Wnt- and β-catenin-responsive gene, mediates radioresistance primarily through suppressing irradiation-induced DNA damage and activating PI3K kinase [139]. Zhang and colleagues [47] reported that ESCC patients with high WISP1 expression had a significantly poorer prognosis compared with those with low WISP1 levels after radiotherapy. The authors further assayed the expression of 94 cancer-related lncRNAs in WISP1-overexpressed EC cells that received radiation, and they identified 14 upregulated lncRNAs and 5 downregulated lncRNAs. Among these lncRNAs, BOKAS was strongly associated with the irradiation-induced upregulation of WISP1. BOKAS is a natural antisense transcript of BOK, a member of the pro-apoptotic Bcl-2 family. Moreover, the downregulation of BOKAS decreased WISP1 expression and greatly enhanced irradiation-induced DNA damage in EC cells. Taken together, these findings indicate that BOKAS induces radioresistance via promoting the upregulation of WISP1.

## LncRNAs in the regulation of cancer stem cells

CSCs only represent a small portion of cells within a given cancer, but they are believed to be responsible for self-renewal, metastatic ability, tumorigenicity, and therapeutic resistance [140–143]. Although ECSCs play a critical role in EC, only a few lncRNAs have been discovered to be associated with the functions of these cells. As an example, MALAT1 has been demonstrated to be associated with tumor stem regulation in several cancer types [144–146]. A recent study by Wang et al. [147] reported that the downregulation of MALAT1 repressed the cancer stem cell-like traits of ECSS through decreasing the expression of tumor stem genes OCT4 and Nanog.

## Clinical applications of lncRNAs in EC

It is recognized that the delayed diagnosis of EC results in metastasis and recurrence and is therefore a major obstacle for EC therapy. Recent studies have demonstrated that lncRNAs play a vital role in the pathological progression of EC. More importantly, lncRNAs have tissue and cell-type specificity. These patterns make lncRNAs attractive as potential biomarkers for the diagnosis and prognosis of EC (Table 3).

**Table 3:** The potential clinical applications of deregulated lncRNAs in EC

Potential application	LncRNA	Expression	Clinical significance	Sample size	Ref.
Diagnostic biomarker	POU3F3	Up	–	Plasma of 147 ESCC patients and 123 healthy donors	[148]
	Linc00152, CFLAR-AS1, and POU3F3	Up	Poor post-surgery prognosis	Plasma of 205 ESCC patients, 82 esophagus dysplasia patients and 210 healthy donors	[149]
	MIR31HG	Down	TNM stage, lymphatic metastasis, and poorer OS	Plasma of 205 ESCC patients and 39 healthy donors	[153]
Prognostic biomarker	ATB	Up	TNM stage and poor DFS	150 paired ESCC tissues	[51]
	XIST	Up	Shorter DFS and OS	127 paired ESCC tissues	[74]
	AK001796	Up	TNM stages, lymph node metastasis, and shorter OS	50 paired ESCC tissues	[93]
	ZEB1-AS1	Up	Tumor grade, depth of invasion, lymph node metastasis, and shorter DFS and OS	87 paired ESCC tissues	[145]
	MALAT1	Up	Lymphatic invasion, distant metastasis, tumor differentiation, and shorter OS	106 paired ESCC tissues	[147]
	PCAT-1	Up	Lymph node metastasis, TNM stage, and poorer OS	130 paired ESCC tissues	[161]
	NKILA	Down	Tumor size, TNM stage, poor DFS, and OS	137 paired ESCC tissues	[162]
	SPRY4-IT1	Up	Clinical stage and shorter OS	92 paired ESCC tissues	[163]
	ZFAS1	Up	Poor OS	50 paired ESCC tissues	[164]

## Tumor diagnosis

Emerging evidence has demonstrated that early diagnosis and effective intervention improves the survival of EC patients. LncRNAs are involved in EC oncogenesis and progression, and the presence of lncRNAs in the peripheral blood and body fluids of EC patients suggests that lncRNAs could serve as diagnostic biomarkers [89, 90]. Tong et al. [148] analyzed the levels of ten lncRNAs in 48 plasma samples and found that POU class 3 homeobox 3 (POU3F3), HNF1A-AS1, and SPRY4-IT1 were markedly higher in ESCC patients compared to healthy controls. In addition, in a cohort of 147 ESCC patients and 123 healthy volunteers, the receiver operating characteristics (ROC) curves demonstrated a strong separation between ESCC patients and healthy volunteers, with an area under the curve (AUC) of 0.842 (95% CI 0.794–0.890; *p* < 0.001) for POU3F3, with a 72.8% sensitivity and 89.4% specificity. In another study, Hu and colleagues [149] found that Linc00152, CASP8- and FADD-like apoptosis regulator-antisense 1 (CFLAR-AS1), and POU3F3 were significantly upregulated in a large cohort of 205 ESCC patients and 82 esophagus dysplasia patients compared to 210 healthy controls, with an AUC of 0.698, 0.651, and 0.584, respectively. The merged AUCs of the three lncRNAs were 0.765, while the AUC increased to 0.955 after merging the three factors with CEA. The circulating levels of the three lncRNAs were associated with poor postsurgery prognoses of ESCC patients in Kaplan–Meier curves. The authors also demonstrated the stability of the lncRNAs that were expressed in the human plasma, which is a crucial prerequisite for a biomarker. HOTAIR was shown to be significantly upregulated in ESCC tissues [150, 151]. A recent study demonstrated that the expression level of HOTAIR in the serum of ESCC patients (*n* = 50) was significantly higher compared to healthy controls (*n* = 20), with an AUC of 0.793 (95% CI 0.692 to 0.895, *P* < 0.01) and optimal cutoff values of 0.094 (sensitivity 56.0%, specificity 90.0%) [152]. In addition, the serum level of HOTAIR was positively correlated with the distant metastasis and TNM stage. MicroRNA-31 host gene (MIR31HG) is another EC-related lncRNA that is significantly upregulated in EC tissues compared to the adjacent normal tissues, as well as in ESCC plasma, compared to the healthy individuals [153]. In addition, plasma MIR31HG was found to differentiate between ESCC patients and healthy individuals by AUC analysis (95% CI 0.656 to 0.841, *P* < 0.01). These findings indicated that POU3F3, HOTAIR, and MIR31HG may be potential biomarkers for EC diagnosis. However, given that these lncRNAs have been shown to be dysregulated in cancers other than EC [154–160], they may best serve as effective diagnostic biomarkers in EC in combination with other variables.

## Tumor prognosis

In recent years, great advances have been made in research into lncRNA-related prognostic biomarkers. The aberrant expression of several lncRNAs has been significantly associated with EC prognosis and may serve as potential prognostic predictors.

The expression of prostate cancer-associated ncRNA transcript 1 (PCAT-1) was markedly upregulated in 130 cancerous tissues compared to matched noncancerous tissues in ESCC [161]. High expression levels of PCAT-1 have been correlated with the depth of tumor invasion, lymph node metastasis, and TNM stage. Kaplan–Meier analysis has revealed that patients in the high PCAT-1 group (*n* = 65) had shorter survival times compared with those in the low PCAT-1 group (*n* = 39). The expression of lncRNA ZEB1-AS1 (ZEB1 antisense 1) was significantly upregulated in 87 ESCC tissues compared to the adjacent noncancerous tissues and was significantly associated with the depth of invasion and lymph node metastasis [145]. In addition, from the Kaplan–Meier survival curves, it was observed that the 5-year overall survival (OS) and disease-free survival (DFS) of ESCC patients with high levels of ZEB1-AS1 were shorter compared with those with low levels of ZEB1-AS1.

Additionally, the dysregulation of lncRNAs ATB [51], XIST [74], AK001796 [93], MALAT1 [147], nuclear transcription factor NF-κB interacting lncRNA (NKILA) [162], SPRY4-IT1 [163], and zinc finger antisense 1 (ZFAS1) [164] has also been demonstrated to be markedly associated with advanced lymph node metastasis, aggressive TNM stage, and shorter survival time. These lncRNAs may also serve as potential prognostic biomarkers for EC.

## Conclusions

EC is the eighth most frequently diagnosed malignancy worldwide. Due to typically late diagnoses at the advanced stage, combined with lymphatic metastasis, the prognosis of EC patients is poor. Despite advancements in surgery, chemo- and radiotherapy treatment over the past decades, few encouraging improvements in the 5-year OS rate of EC patients have been achieved. Moreover, the molecular mechanisms underlying EC tumorigenesis and development are still elusive. Hence, a comprehensive understanding of the molecular pathogenesis and identification of potential biomarkers of this disease are urgently needed. It is now recognized that aberrant expression of lncRNAs is a crucial determinant for human cancer. In this review, we have summarized the molecular mechanisms of lncRNAs and how they function in EC by localizing to the chromatin and interacting with proteins and RNAs. Uncovering the underlying mechanisms of lncRNAs may help us to understand the pathogenesis and progress of EC, including cell apoptosis, proliferation, migration, stemness, and therapy resistance. Furthermore, lncRNAs have the potential to serve as promising biomarkers for diagnosing EC and predicting prognosis and relapse, and they may even be novel attractive targets for clinical therapy of EC. However, there remain significant gaps in our understanding of the functions of lncRNAs in EC; these gaps must be bridged before lncRNAs can be used in clinical practice.

## Abbreviations

3′-UTR: 3′-Untranslated regions
AFAP1-AS1: AFAP1-antisense 1
AS: Alternative splicing
ATB: Activated by transforming growth factor β
AUC: Area under the curve
CASC2: Cancer susceptibility candidate 2
CASC9: Cancer susceptibility candidate 9
CBP: CREB-binding protein
CCAT1: Colon cancer-associated transcript-1
CDKN2B-AS1: Cyclin-dependent kinase inhibitor 2B-antisense 1
ceRNAs: Competing endogenous RNAs
CFLAR-AS1: CASP8 and FADD-like apoptosis regulator-antisense 1
ChIRP: Chromatin isolation by RNA purification
DFS: Disease-free survival
EAC: Esophageal adenocarcinoma
EC: Esophageal cancer
EMT: Epithelial–mesenchymal transition
ESCC: Esophageal squamous cell carcinoma
EZR-AS1: EZR-antisense 1
GHET1: Gastric carcinoma highly expressed transcript 1
HNF1A-AS1: HNF1A-antisense 1
HOTAIR: HOX transcript antisense RNA
HOTTIP: HOXA transcript at the distal tip
LET: Low Expression in Tumor
lncRNAs: Long non-coding RNAs
LUCAT1: Lung cancer-associated transcript 1
MALAT1: Metastasis associated in lung adenocarcinoma transcript 1
MIR31HG: MicroRNA-31 host gene
MREs: miRNA response elements
NEAT1: Nuclear paraspeckle assembly transcript 1
NKILA: Nuclear transcription factor NF-κB interacting lncRNA
OS: Overall survival
PCAT-1: Prostate cancer-associated ncRNA transcript 1
POU3F3: POU class 3 homeobox 3
POU6F2-AS2: POU6F2-antisense 2
PVT1: Plasmacytoma variant translocation 1
ROC: Receiver operating characteristics
SBF2-AS1: SET-binding factor 2 antisense RNA 1
SNHG1: Small nucleolar RNA host gene 1
SNHG16: Small nucleolar RNA host gene 16
SPRY4-IT1: Sprouty4-Intron 1
TF: Transcription factor
TP73-AS1: P73 antisense RNA 1T
TTN-AS1: TTN-antisense 1
TUSC7: Tumor suppressor candidate 7
UCA1: Urothelial carcinoma associated 1
XIST: X-inactive specific transcript
ZEB1-AS: ZEB1 antisense 1
ZFAS1: Zinc finger antisense 1
